# Win statistics applied to registry-based randomized clinical trials

**DOI:** 10.1186/s13063-026-09598-3

**Published:** 2026-03-07

**Authors:** Rebecca Rylance, Philippe Wagner, Matthias Götberg, Moman A. Mohammad, Robin Hofmann, Ole Fröbert, Stefan James, David Erlinge

**Affiliations:** 1 Department of Cardiology, Clinical Sciences, Lund University, Skåne University Hospital, Lund, 221 85 Sweden; 2 Centre for Clinical Research, Uppsala University, Västerås, Sweden; 3 Department of Clinical Science and Education, Karolinska Institute, Södersjukhuset, Stockholm, Sweden; 4 Department of Cardiology, Faculty of Health, Örebro University, Örebro, Sweden; 5 Department of Clinical Medicine, Aarhus University Health, Aarhus, Denmark; 6 Department of Medical Sciences, Cardiology, Uppsala University, Uppsala, Sweden

**Keywords:** Win ratio, Win odds, Composite endpoints, Hierarchical composite endpoints, Cox ph model, Registry-based data

## Abstract

**Background:**

Win statistics offer an alternative approach to clinical trials that use survival analysis to analyze composite endpoints. Our objective was to re-analyze data from previously published registry-based randomized controlled trials that produced hazard ratios using win statistics to evaluate the correspondence between them. Good correspondence was defined as both results being positive, negative, or neutral. Win statistics were calculated for these trials to encourage transparency, scientific rigor, and possibly validate results.

**Methods:**

The win ratio ordered events hierarchically by clinical importance for each trial, with all-cause death regarded as most severe, followed by acute myocardial infarction. Further components, i.e., other endpoints, were added subsequently to the hierarchy. Each patient in the treatment group was compared with each patient in the control arm in hierarchical order. The total number of wins for each category in the treatment group was added and divided by the total number of wins in the control group. Win odds were calculated as an extension, which incorporate ties into the calculation.

**Results:**

The results using win statistics showed good correspondence to the previously reported hazard ratios with their composite endpoints: for the TASTE trial, the results were neutral for both the hazard ratio and win odds. The hazard ratio was 0.86 (0.67–1.10), and the win odds were 1.02 (0.99–1.04). Similar results were found for the iFR-SWEDEHEART, DETO2X-AMI, and VALIDATE trials. The IAMI trial showed better results for the vaccinated group compared to the placebo for both the hazard ratio and win odds.

**Conclusions:**

Win statistics offer an alternative approach to traditional survival analysis by harnessing multiple events hierarchically by clinical importance. Win statistics also offer the potential to evaluate a broader range of clinical endpoints, providing a more rounded perspective of treatment efficacy, an important consideration when designing future randomized controlled trials. This is the first multi-registry-based controlled trial reanalysis using win statistics.

**Supplementary Information:**

The online version contains supplementary material available at 10.1186/s13063-026-09598-3.

## Introduction

The use of traditional composite endpoints is common practice in cardiovascular research for several reasons [1]. They require a smaller sample size, evaluate a net effect, and avoid bias in the presence of competing risks. However, composite endpoints also have certain drawbacks. In time-to-event analyses with composite endpoints, only the time to the first event is counted even though the event that occurs first may not be the most clinically relevant [2, 3]. With this approach, vital information may be lost or misinterpreted.

In contrast, the win ratio (WR), which was introduced in 2011, compares the number of wins in one group to the number of wins in the other group, prioritizing events by clinical importance [4–6]. The win odds (WO) extend the WR to incorporate ties into the calculation [7]. Together, they form the win statistics, two flexible analytic tools where all event types may be incorporated, thereby increasing power to detect a treatment difference.

Win statistics adhere to a hierarchy and summarize wins, losses, and ties in order unlike composite endpoints, which count the time to the first event. Flexible endpoints such as quality-of-life scores may be added to win statistics as well, which is not possible with composite endpoints. Cox proportional (ph) hazards models compare hazard rates over time between groups while win statistics compare the summed probabilities of clinically prioritized wins between groups.

A recent Swedish-UK registry-based randomized controlled trial (RRCT), Dapagliflozin in Myocardial infarction without Diabetes or Heart failure, features the WR as its primary outcome in a RRCT. This trial ranks non-cardiovascular death, heart failure event, non-fatal acute myocardial infarction (AMI), atrial fibrillation/flutter event, new diagnosis of type 2 diabetes, NYHA Functional Classification at the last trial visit, and body weight decrease of 5% or more from baseline to the last trial visit in its hierarchical composite endpoints [8].

The current study fills a gap in the literature by reanalyzing previous published findings with win statistics. RRCTs generate rich longitudinal registry outcomes that are often not fully captured by first-event Cox ph models. Win statistics provide an opportunity to re-evaluate total patient burden. Re-analyzing data also encourages transparency, scientific rigor, and may validate results.

The primary objective of this study is to re-evaluate five RRCTs using win statistics to assess the correspondence between the hazard ratios (HR) and win statistics [9, 10]. Good correspondence refers to the confidence intervals (CIs) for HR and win statistics in the same study. These are defined as both CIs for HR and win statistics being positive, meaning the CI for the HR being less than one while the CI for the win statistics being greater than one, or both negative, meaning the CI for the HR being greater than one while the CI for the win statistics being less than one, or both neutral, which means both CIs crossing one. Directional correspondence was preferred over concordance in magnitude since quantifying concordance in magnitude from two different methods would be impractical.

## Methods

### All RRCTs included in the analysis were conducted in Sweden

Summary details of each trial can be found in Table 1. The Thrombus Aspiration during ST-Segment Elevation Myocardial Infarction (TASTE) trial determined no statistically significant differences at 30 days or 1 year for thrombus aspiration and percutaneous coronary intervention (PCI) versus PCI alone for the primary endpoint of all-cause death [11, 12]. The Instantaneous Wave-free Ratio (iFR) versus Fractional Flow Reserve (FFR) in Patients with Stable Angina Pectoris or Acute Coronary Syndrome trial (iFR-SWEDEHEART—Swedish Web-Based System for Enhancement and Development of Evidence-Based Care in Heart Disease Evaluated According to Recommended Therapies) showed non-inferiority of IFR-guided revascularization compared to FFR for the composite endpoint of all-cause death, AMI, or unplanned revascularization at 12 months [13]. The Determination of the Role of Oxygen in Suspected AMI (DETO2X-AMI) trial found no statistically significant differences in all-cause death at 365 days between supplemental oxygen and ambient air in patients with suspected AMI [14]. The VALIDATE-SWEDEHEART Bivalirudin versus Heparin in ST-Segment (STEMI) and Non–ST-Segment Elevation Myocardial Infarction (NSTEMI) in Patients on Modern Antiplatelet Therapy in SWEDEHEART trial evaluated bivalirudin and heparin with no statistically significant differences for the composite endpoint of all-cause death, AMI, or major bleeding at 180 days [15]. The IAMI (Influenza Vaccination After Myocardial Infarction) trial tested whether AMI patients benefited from inactivated influenza vaccine or saline placebo at 12 months. The composite endpoint of all-cause death, myocardial infarction, or stent thrombosis was statistically significantly lower in the active treatment group [16].

**Table 1:** Summary of findings of 5 RCTs

Name of study	TASTE	iFR-SWEDEHEART	DETO2X-AMI	VALIDATE	IAMI
What it tested, type of AMI, number of patients	This trial tested the superiority of thrombus aspirin + PCI vs. PCI alone in STEMI patients. *n* = 7244	The noninferiority of iFR vs. FFR by a margin of 3.2 in stable angina, unstable angina NSTEMI patients. *n* = 2037	The superiority of oxygen therapy vs. ambient air in STEMI and NSTEMI patients. *n* = 6629	The superiority of bivalirudin vs. heparin in STEMI and NSTEMI patients. *n* = 6006	The superiority of vaccine vs. placebo, in STEMI and NSTEMI patients. *n* = 2571
Endpoint	All-cause death at 30 days	All-cause death, AMI, or unplanned revascularization at 12 months	All-cause death at 365 days	All-cause death, AMI, or bleeding at 180 days	All-cause death, AMI, or stent thrombosis at 12 months
Method	Cox ph model	Test for inferiority	Cox ph model	Cox ph model	Cox ph model

*AMI *acute myocardial infarction, *Cox ph model *Cox proportional hazards model

All the original trials followed prespecified protocols. Some of the trials adjusted for additional variables. When the Cox ph assumption was not met in the IAMI trial, time-dependent HRs and stratification variables were implemented. The other trials reported *p* values from the predefined primary analysis regardless of model fit, although logistic regression models supported the primary analysis or were treated as sensitivity analyses.

### Win statistics

Win statistics, both the WR and WO, used the unmatched approach. The unmatched approach compared every person in the treatment group to every person in the control group [4]. This is motivated in the randomized setting because baseline risk is balanced because of the randomization [17]. When the win ratio was first developed, confidence intervals for the unmatched approach were derived from bootstrapping, but then a closed-form variance estimator was developed [18]. With the matched approach, patients with similar baseline risk are paired. Negatives with this approach include the possibility of not finding matches for every patient, which leads to a loss of observations as well as the subjectiveness of the matching process [5, 19]. Confidence intervals for the matched approach may be derived from bootstrapping or *U*-statistics.

The hierarchy included the original main endpoints from the trials first, then the severity, availability, and relevance for coronary intervention trials were chosen with severity being the most decisive [20]. Death is more severe than myocardial infarction. Stent thrombosis has a worse outcome than any revascularization. For all 5 trials, the hierarchy started with death and AMI. Additional components of the hierarchical composite endpoint were added, followed by secondary endpoints, and lastly, other available endpoints, which were only included if clinically relevant. A complete list of hierarchical outcomes priorities was established for each RRCT (Table 2).

**Table 2:** List of clinical priorities by RCTs

TASTE	iFR-SWEDEHEART	DETO2X-AMI	VALIDATE	IAMI
Death	Death	Death	Death	Death
Acute myocardial infarction	Acute myocardial infarction	Acute myocardial infarction	Acute myocardial infarction	Acute myocardial infarction
Stent thrombosis	Unplanned revascularization	Heart failure	Bleeding	Stent thrombosis
Target vessel revascularization	Target lesion revascularization	Stent thrombosis	Stent thrombosis	
Restenosis	Restenosis	Cardiogenic shock	Stroke	

Win statistics were conducted for STEMI and NSTEMI separately when data were available (Supplement).

In each trial, patient-to-patient pairs were formed and within each pair, component outcomes were assessed in descending order of clinical importance until a superior outcome was identified in one of the pairs [21]. Starting with death, if both patients in a pair died, the patient that died later counted as a win. Conversely, in a pair where one patient died and the other suffered an AMI, the patient with the AMI counted as a win. Should the comparison across all listed outcomes result in no events, the pair was classified as a tie. The total number of wins in each category for the treatment group was summed and divided by the total number of wins for the control group. A WR of 1.5 indicated that there were 50% more wins, while a WR of 0.5 indicated 50% fewer wins [4]. The win ratio can be converted to a probability, *p* = Win ratio/(Win ratio + 1) [22]. A win ratio of 1.5 means that the patient has a probability 0.6 of success on the treatment and a probability of 0.4 of success on placebo. The larger the win ratio, the more evidence of an effect. Although, much like traditional composite endpoints, the hierarchical endpoint that is driving the results must be taken into consideration.

The WO method was included as an extension of the WR approach, incorporating ties [7]. Ties were incorporated by adding half a win to the numerator and half a loss to the denominator of the WR. Many ties indicated similarity between treatment and control, and this is accounted for in the WO. If a study had no ties, the WR equaled the WO, thereby yielding identical results. However, both win statistics tested the same null hypothesis of equal win probabilities [9].

According to ICH E9(R1) guidelines, there are defined estimand strategies that direct clinical questions [23]. An estimand is defined as a specific explanation of the treatment effect explaining the clinical question posed by the study aim. Win statistics apply a composite strategy where the treatment effect is measured as a summation of wins compared to losses and the intercurrent event is incorporated into the definition.

### Prespecification and post hoc decisions

The hierarchies were prespecified for this trial, although the original trial results were not blinded during the planning of this study. However, sensitivity analysis including only death and acute myocardial infarction as well as reordering the other endpoints was conducted (Supplement). No multiplicity adjustments were prespecified because of the exploratory nature of this study. No sample size calculations were required since the sample size was predetermined from the original trial data. Clean data was provided from the primary investigator of each trial and data quality checks were done for data consistency and missingness. Minor data management was performed. For example, in STATA, time variables cannot be 0, so these were altered to 0.001 and to be able to calculate the win odds in R, variables needed to be renamed and reordered as specified in the WINS package.

All tests were two-sided with 95% confidence intervals. Win statistics were calculated using both R 4.5.1 with the WINS package and STATA 17 with the winratio command with a usual runtime of under 5 min. Example code is provided (Supplement).

## Results

Across the five trials, win statistics were directionally consistent with HRs in all cases; effect magnitudes differed because of the high percentage of ties.

**Fig. 1:**
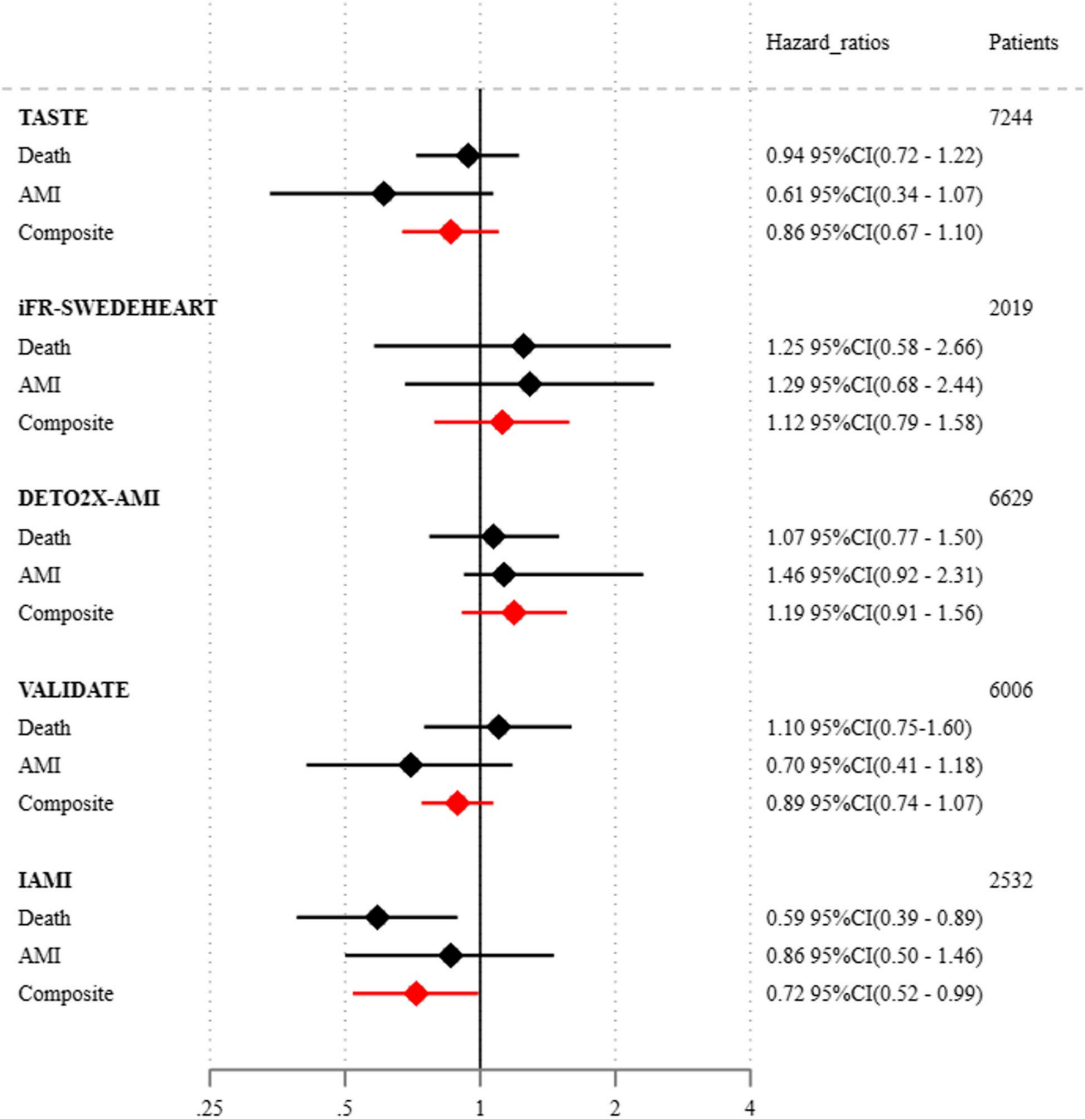
Hazard ratios for iFR-SWEDEHEART and IAMI at 12 months and 30 days for the other trials

The original composite trial results and the hierarchical composite endpoints from the present study are shown in Table 3. HRs presented with 95%CI for death, AMI, and the combined composite endpoints of death and AMI (Fig. 1).

**Table 3:** Original composite results and win statistics by RCTs

	HR-original trial	*p* value	WR	*p* value	WO	*p* value	% ties
TASTE							
30 days	0.86 (0.67–1.10)	0.23	1.18 (0.97–1.45)	0.10	1.02 (0.99–1.04)	0.13	89.7%
1 year	0.94 (0.80–1.11)	0.48	1.11 (0.97–1.26)	0.14	1.02 (0.99–1.05)	0.15	77.7%
iFR-SWEDEHEART							
12 months	1.12 (0.79–1.58)	0.53	0.84 (0.60–1.18)	0.33	0.98 (0.94–1.02)	0.32	87.1%
DETO2X-AMI							
30 days	1.19 (0.91–1.56)	0.21	0.92 (0.73–1.17)	0.52	1.00 (0.98–1.01)	0.59	91.9%
365 days	1.03 (0.87 1.22)	0.70	0.96 (0.83–1.12)	0.63	0.99 (0.97–1.03)	0.71	79.5%
VALIDATE							
30 days	0.89 (0.74–1.07)	0.21	1.09 (0.92–1.29)	0.34	1.01 (0.98–1.04)	0.44	83.5%
180 days	0.96 (0.83–1.10)	0.54	1.03 (0.90–1.18)	0.68	1.01 (0.97–1.04)	0.76	75.0%
IAMI							
12 months	0.72 (0.52–0.99)	0.04	1.40 (1.02–1.92)	0.04	1.04 (1.00–1.08)	0.04	88.0%

All confidence intervals are 95%CIs

*HR *hazard ratios, *WR *win ratios, *WO *win odds

There was no missing data.

**Fig. 2:**
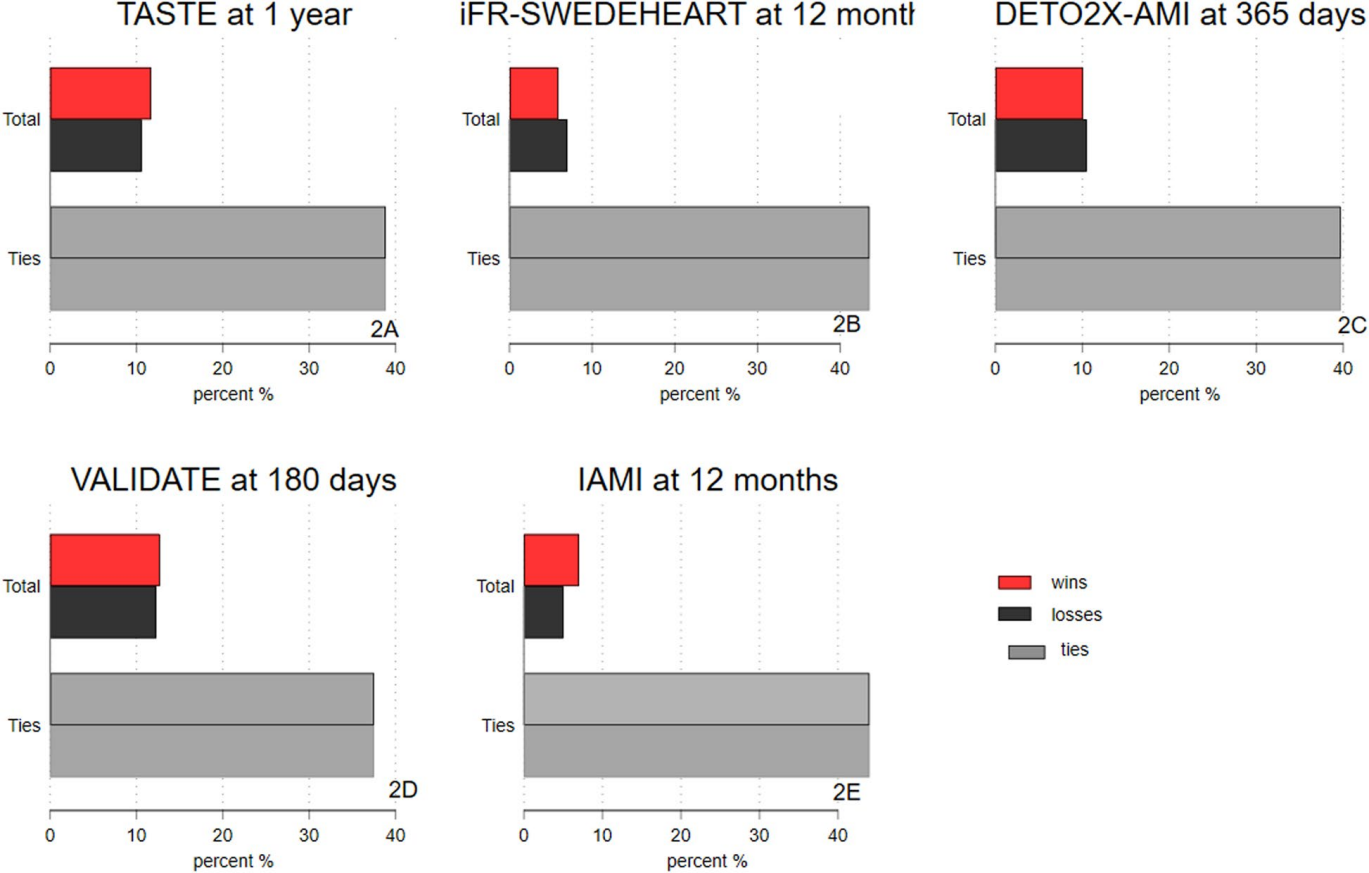
Breakdown of total wins, losses, and ties in percent for each trial (Total + Ties equals 100%). Wins represent an advantage for treatment over control

In the TASTE trial, the HR for the composite outcome at 30 days was 0.86 (0.67–1.10). The WR was 1.18 (0.97–1.45) at 30 days and the WO 1.02 (0.99–1.04). The proportion of ties was 89.7% (Fig. 2A). A WR of 1.18 may appear impactful, but a WO 1.02 suggests a negligible difference. The WR magnitude can be inflated when ties dominate.

For the iFR-SWEDEHEART, composite outcome at 12 months was HR 1.12 (0.79–1.58). The WR was 0.84 (0.60–1.18) and the WO 0.98 (0.94–1.02) at 12 months. The proportion of ties was 87.1% (Fig. 2B).

In the DETO2X-AMI trial the results for the composite outcome at 30 days was HR 1.19 (0.91–1.56). The WR was 0.92 (0.73–1.17) and WO of 1.00 (0.98–1.01). The proportion of ties was 91.9% (Fig. 2C).

For the VALIDATE trial, the composite outcome at 30 days was HR 0.89 (0.74–1.07). The WR was 1.09 (0.92–1.29) and WO of 1.01 (0.98–1.04) at 30 days. The proportion of ties was 83.5% (Fig. 2D).

**Fig. 3:**
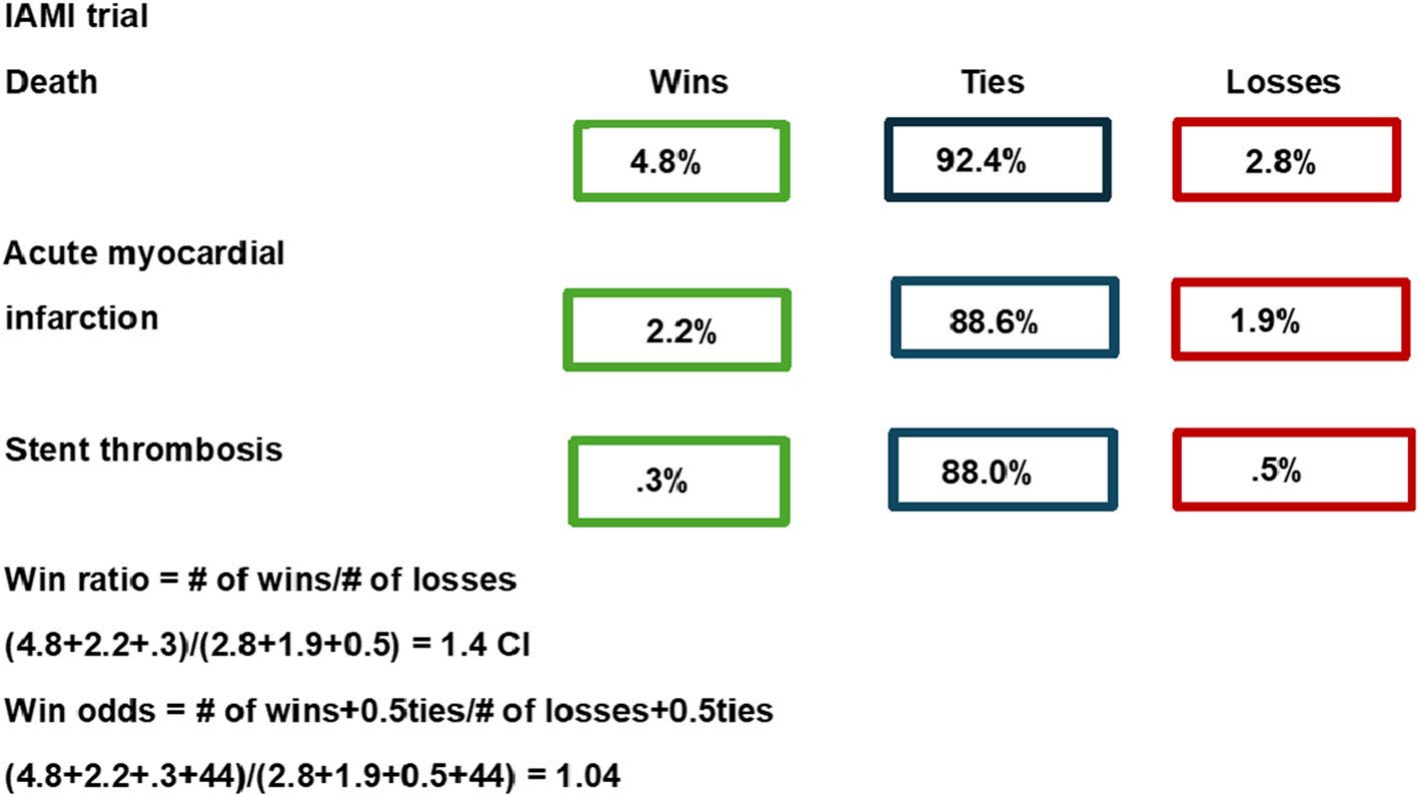
A methodological overview figure of the IAMI trial

For the IAMI trial, statistically significant differences were found for the composite at 12 months, HR 0.72 (0.52–0.99). Similarly, the WR was 1.40 (1.02–1.92) and a WO of 1.04 (1.00–1.08). The proportion of ties was 88.0%. In the NSTEMI group, the WR was 1.69 (1.12–2.54) and the WO 1.09 (1.02–1.16), while in the STEMI population, WR 1.12 (0.67–1.88) and WO 1.01 (0.97–1.05) (Fig. 2E). A methodological overview figure has been provided for the IAMI trial (Fig. 3).

The sensitivity analysis found no deviations from the original hierarchies. These results are presented in the supplement.

**Fig. 4:**
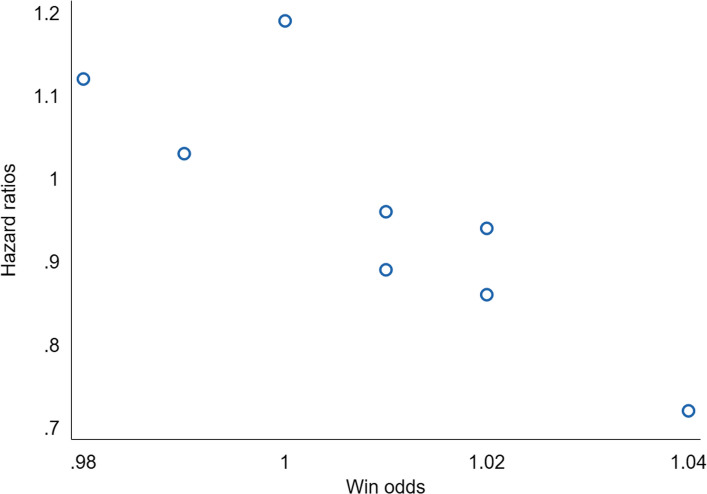
A scatterplot of hazard ratios and win odds

Figure 4 shows a scatterplot of hazard ratios and win odds.

Figure 2F–J show total wins and losses, individual endpoints, and ties by trial (Supplement). For demographics, please see original publications [11–15].

## Discussion

Win statistics produce results with good correspondence to the original HR-based analyses across all five RRCTs by validating previous results, which means the conclusions are compatible under different statistical questions. Although they do not replace Cox ph models, they contribute a viable complementary approach. In the IAMI trial, the results show an increase in wins in the influenza vaccinated group, which can be attributed to the NSTEMI population, which agrees with a previous subgroup analysis [24]. This may be attributed to different inflammatory mechanisms between the two types of AMIs.

The large proportion of ties between treatment groups reflects the real-world similarity in outcomes between treatment groups, mitigating a treatment effect. Values close to one represent negligible clinical differences. Many ties also indicate low-event rates, shorter follow-up, and balanced treatment effects. Furthermore, substantial ties distort the magnitude of the WR, which hampers interpretability and muddles clinical signals. Many ties along with large sample sizes produce extremely narrow CIs in the WO. Narrow confidence intervals in the presence of many ties should not be over-interpreted as a signal of extreme precision.

In the Prospective angiotensin receptor–neprilysin inhibitor vs. angiotensin-converting enzyme inhibitor trial to determine superiority in reducing heart failure events after myocardial infarction, trial randomized patients to sacubitril/valsartan or ramipril [25, 26]. The composite endpoint HR is neutral at 0.90 (0.78–1.04), while the WR is positive at 1.17 (1.03–1.33), indicating a lack of correspondence. However, the WO statistic is 1.05, whose magnitude is greatly attenuated from the WR because of the large number of ties suggesting that in the presence of many ties, the WO is a must [17].

Otherwise, if the number of ties is negligible like in the Semaglutide in patients with heart failure with preserved ejection fraction and obesity trial with only 5.1% ties, the stratified WR is 1.72 (1.37–2.15) and reflects nearly the entire population [27].

The value of the WR over traditional survival analysis is the varied components like in the Semaglutide trial, which included softer endpoints such as change in Kansas City Cardiomyopathy Questionnaire–Overall Summary (KCCQ-OS) score.

Another innovative application of win statistics is the Design and analysis of studies based on hierarchical composite endpoints: insights from the DARE-19 trial that compare the effects of dapagliflozin with placebo in COVID-19 patients [28]. Patients are placed into five categories, with each category building on the previous one, and within each of these categories, patients are ranked by the timing of their event.

Even when they confirm rather than contradict existing methods, the strength of win statistics is their flexibility to include a variety of endpoints, which increase power and overall understanding of treatment efficacy. Win statistics are more suitable to Cox ph models when there is an interest in diverse endpoints. In practice, when patients are compared with traditional methods like Cox ph models, only a few very hard endpoints such as death or AMI are included. With win statistics, endpoints that may be of importance to the patient are included such as hospitalizations.

Time-to-first event analysis may dilute treatment effects, but win statistics incorporate many event types, and in registry settings, multiple endpoints may be available. Last-event-assisted win ratio may be beneficial for diseases with recurrent events because it counts repeated events and tests if a treatment continually produces more wins over time.

The future of win statistics in cardiovascular trials will depend on streamlining which components capture disease burden. Trialists and clinicians should consider including win statistics as prespecified exploratory analysis derived from steering groups, Delphi panels with input from patient-reported importance rankings. Clinical severity and patient-perceived burden may not always be aligned, but a consensus should be established as the hierarchical order places judgment on the importance of outcomes with most hierarchies prioritizing clinician- and trialist-driven severity judgments. Overmatching, which reduces efficiency, can be avoided by not including too many low-severity endpoints. After the hard endpoints like death and AMI, trials should include unambiguous endpoints that contribute vital information to the patient. Global rank methods as well as restricted mean survival may also be potential methods to evaluate disease burden.

The use of combining RRCT with win statistics can also be used for other disease areas such as oncology, neurology, and chronic inflammatory disease as long as the registry has high coverage and meaningful secondary endpoints are registered.

A limitation of this study is the post hoc nature of the design as well as the non-blinding of the original results, which may have led to bias.

Another limitation is that information on stroke date is not available for TASTE. If this information had been available, it would most likely have made the results of the win statistics more neutral since the TOTAL trial (thrombectomy with PCI versus PCI alone in patients with STEMI undergoing primary PCI) found an increase in stroke risk in the thrombectomy group at 30 days [29].

Although beyond the scope of this paper, trial heterogeneity makes it difficult to compare win statistics across trials. Patients had varied cardiovascular profiles as well as diverse hierarchical endpoints that were included in the win statistics.

Registry-based data is subject to misclassification, incomplete data, and time-delayed entry that is an integral part of registry-based data [30]. Since trials may span several countries, data synchronization should be standardized. Data from Sweden is considered high-quality with regular data audits [31]. Outside of Sweden, data quality may suffer from a lack of coordination across healthcare systems, which may impact external validity [32]. Transparency and open scientific communication can be improved by more studies providing access to code snippets and statistical analysis plans.

## Conclusions

This study is unique because it is one of the few studies to re-examine RRCTs using win statistics encouraging the re-analyzation of RRCTs to either support or refute previous findings. Prospective inclusion of win statistics in RRCT protocols may enhance methodological robustness. It is important to present the WO as the primary estimand over the WR when the percentage of ties > 5%. Unlike the WR, the WO includes all observations; therefore, the WO describes the entire population, not only those that are classified as a win or a loss. In the future, CONSORT and STROBE guidelines could be expanded to introduce a concrete framework for how to order hierarchical endpoints.

## Supplementary Information

Supplementary Material 1.

Supplementary Material 2.

## Data Availability

Data are available upon reasonable request from the primary investigator of each trial.

## Abbreviations

RRCTs: Registry-based randomized controlled trials
AMI: Acute myocardial infarction
WR: Win ratio
WO: Win odds
TASTE: Thrombus Aspiration during ST-Segment Elevation Myocardial Infarction
STEMI: ST-segment elevation myocardial infarction
NSTEMI: Non-ST-segment elevation myocardial infarction
PCI: Percutaneous coronary intervention
iFR: Instantaneous wave-free ratio
FFR: Fractional flow reserve
SWEDEHEART: Swedish Web-Based System for Enhancement and Development of Evidence-Based Care in Heart Disease Evaluated According to Recommended Therapies
DETO2X-AMI: The Determination of the Role of Oxygen in Suspected AMI trial
the VALIDATE-SWEDEHEART trial: Bivalirudin versus heparin in non-ST and ST-segment elevation myocardial infarction—a RRCT in the SWEDEHEART registry
IAMI trial: Influenza Vaccination After Myocardial Infarction
