# Supplementary material for: Win statistics applied to registry-based randomized clinical trials
Source: Trials. 2026 Mar 7;27:279. doi: 10.1186/s13063-026-09598-3 (PMC13063660; doi:10.1186/s13063-026-09598-3)
Supplement: Supplementary file 1 — Supplementary Material 1. [file 13063_2026_9598_MOESM1_ESM.docx]

**Table 4-Win statistics by STEMI/NSTEMI**

| **VALIDATE-30 days** | **Win ratio** | **p-value** | **Win odds** | **p-value** |
| --- | --- | --- | --- | --- |
| STEMI | 1.08 95%CI(0.86-1.37) | 0.49 | 1.01 95%CI(0.97-1.05) | 0.61 |
| NSTEMI | 1.09 95%CI(0.84-1.41) | 0.51 | 1.01 95%CI(0.97-1.05) | 0.57 |
| **VALIDATE-180 days** | 1.03 95%CI(0.90-1.18) | 0.68 | 1.01 95%CI(0.97-1.04) | 0.76 |
| STEMI | 1.05 95%CI(0.87-1.27) | 0.62 | 1.01 95%CI(0.96-1.06) | 0.68 |
| NSTEMI | 1.01 95%CI(0.82-1.23) | 0.95 | 1.00 95%CI(0.95-1.05) | 0.99 |
| **IAMI-12 months** | **Win ratio** | **p-value** | **Win odds** | **p-value** |
| STEMI | 1.12 95%CI(0.67-1.88) | 0.67 | 1.01 95%CI(0.97-1.05) | 0.67 |
| NSTEMI | 1.69 95%CI(1.12-2.54) | 0.01 | 1.09 95%CI(1.02-1.16) | 0.01 |

**Sensitivity analysis of win ratios by admitting or changing hierarchical endpoints**

|  | TASTE  1 year | IFR | DETO2X-AMI | VALIDATE | IAMI 12 months |
| --- | --- | --- | --- | --- | --- |
| Death & AMI  only | 1.06 (0.90 1.24) | 0.77 (0.47 1.27) | 0.96 (0.81 1.13) | 1.00 (0.71 1.40) | 1.48 (1.07 2.05) |
| Switching 2^nd^ & 3^rd^ endpoint | 1.11 (0.97 1.26) | 0.85 (0.60 1.19) | 0.97 (0.83 1.12) | 1.08 (0.91 1.29) | 1.40 (1.02 1.91) |
| Switching 3rd & 4^th^ endpoint | 1.11 (0.97 1.26) | 0.84 (0.60 1.18) | 0.96 (0.83 1.12) | 1.09 (0.91 1.29) | n.a. |

Figure 2F-2J Breakdown of total wins, losses, individual endpoints and ties in percent (Total + Ties equals 100%)


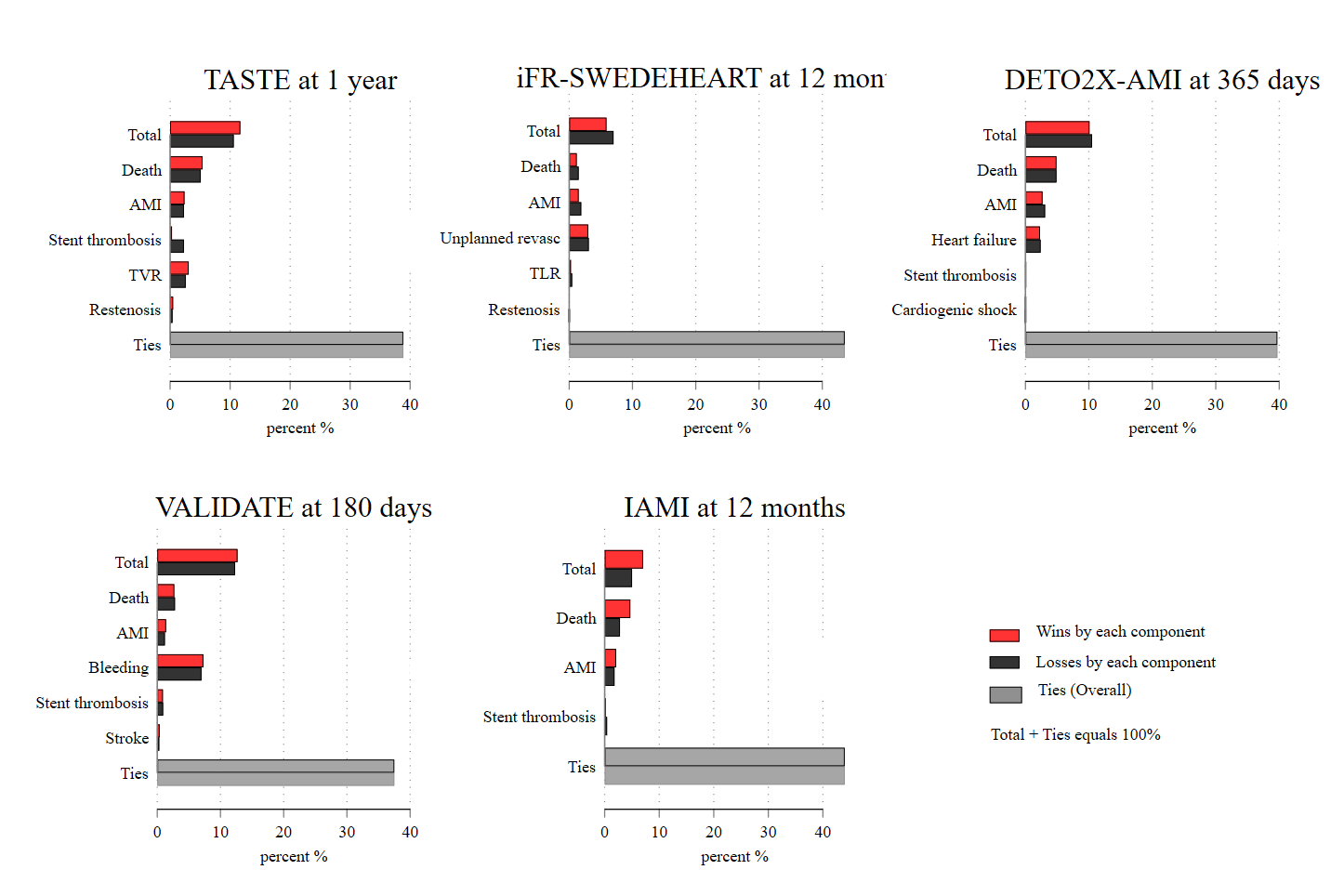


**Scatterplots of hazard ratios and win ratios for each trial**

**
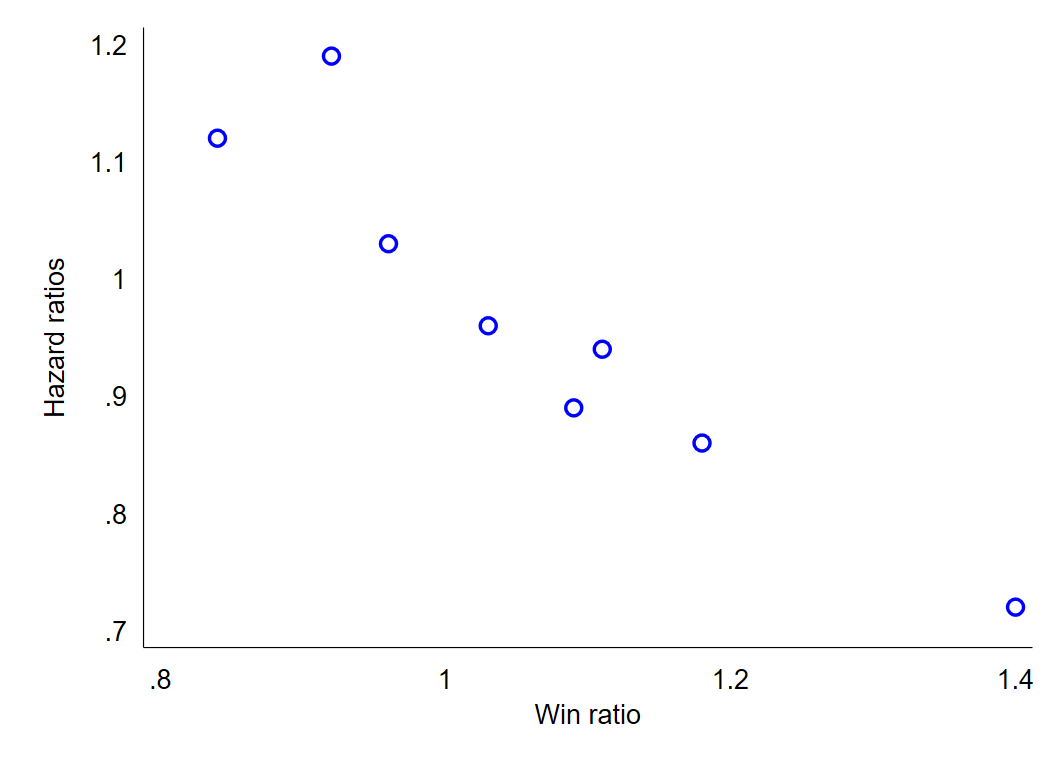
**

**Example code:**

In STATA:

*win ratio is a user-written command so it must be downloaded from ssc

ssc install winratio

*read help file

help winratio

*Description

*keep only relevant variables

keep idvar trtvar dth tf fudth qol

*idvar is a unique identifier variable and may be string or numeric.

*trtvar must be a binary 0 1 numeric variable, where 0 and 1 indicate the control and intervention groups respectively.

*winratio is the command name, followed by the idvar (ID variable) trtvar (Treatment variable), outcomes (outcome variables in order of importance)

*dth, binary time-to-event variable, fudth containing the event time or censoring time

*quality-of-life outcome variables are also possible. in this example qol is a continuous variable, and higher values are better.

winratio idvar trtvar, outcomes(dth tf fudth) outcomes(qol c >)

References

• Pocock SJ, Ariti CA, Collier TJ, Wang D. The win ratio: a new approach to the analysis of composite endpoints in clinical trials based on clinical priorities. Eur Heart J 2012;33:176–182.

• Finkelstein DM, Schoenfeld DA. Combining mortality and longitudinal measures in clinical trials. Stat Med 1999;18:1341–1354.

Authors

Tim Collier, tim.collier@lshtm.ac.uk

John Gregson, john.gregson@lshtm.ac.uk

In R:

*An example for time-to-event, #there are more examples in help file with other outcomes

install.packages("WINS")

require(WINS)

#the dataset needs to be prepared with these exact variable names.

#id A vector for the patient id.

#arm A vector for the treatment groups.

#Delta_1 A vector for the event status of the first endpoint (1=event, 0=censored).

#Delta_2 A vector for the event status of the second endpoint (1=event, 0=censored).

#Delta_3 A vector for the event status of the third endpoint (1=event, 0=censored).

#Y_1 A vector for the outcome of the first endpoint.

#Y_2 A vector for the outcome of the second endpoint.

#Y_3 A vector for the outcome of the third endpoint.

win.stat(data =dataset, ep_type = "tte", arm.name = c(1,0), tau = 0.1,

Z_t_trt1 = Z_t_trt, Z_t_con1 = Z_t_con, priority = c(1:5), alpha = 0.05, digit = 3,

censoring_adjust = "No", weight = "unstratified", pvalue = "two-sided")

References

• Dong, G., Huang, B., Chang, Y.W., Seifu, Y., Song, J. and Hoaglin, D.C., 2020. The win ratio:

Impact of censoring and follow-up time and use with nonproportional hazards. Pharmaceutical

statistics.

• Dong, G., Mao, L., Huang, B., Gamalo-Siebers, M., Wang, J., Yu, G. and Hoaglin, D.C.,

2020. The inverse-probability-of-censoring weighting (IPCW) adjusted win ratio statistic: an

unbiased estimator in the presence of independent censoring. Journal of biopharmaceutical

statistics.

• Dong, G., Hoaglin, DC., Qiu, J., Matsouaka, RA., Chang, Y., Wang, J., Vandemeulebroecke,

M., 2020. The win ratio: on interpretation and handling of ties. Statistics in Biopharmaceutical Research.

• Dong, G., Huang, B., Wang, D., Verbeeck, J., Wang, J. and Hoaglin, D.C., 2021. Adjusting

win statistics for dependent censoring. Pharmaceutical Statistics.

• Dong, G., Huang, B., Verbeeck, J., Cui, Y., Song, J., Gamalo-Siebers, M., Wang, D., Hoaglin,

D.C., Seifu, Y., Mutze, T. and Kolassa, J., 2022. Win statistics (win ratio, win odds, and net

benefit) can complement one another to show the strength of the treatment effect on time-toevent outcomes. Pharmaceutical Statistics.

• Dong, G., Hoaglin, D.C., Huang, B., Cui, Y., Wang, D., Cheng, Y. and Gamalo-Siebers, M.,

2023. The stratified win statistics (win ratio, win odds, and net benefit). Pharmaceutical

Statistics.

• Finkelstein, D.M. and Schoenfeld, D.A., 1999. Combining mortality and longitudinal measures in clinical trials. Statistics in medicine.

• Finkelstein, D.M. and Schoenfeld, D.A., 2019. Graphing the Win Ratio and its components

over time. Statistics in medicine.

• Luo, X., Tian, H., Mohanty, S. and Tsai, W.Y., 2015. An alternative approach to confidence

interval estimation for the win ratio statistic. Biometrics.

• Pocock, S.J., Ariti, C.A., Collier, T.J. and Wang, D., 2012. The win ratio: a new approach

to the analysis of composite endpoints in clinical trials based on clinical priorities. European

heart journal.

• Wang D, Pocock S., 2016. A win ratio approach to comparing continuous non-normal outcomes in clinical trials. Pharmaceutical Statistics.

• Wang, D., Zheng S., Cui, Y., He, N., Chen, T., Huang, B., 2023. Adjusted win ratio using

inverse probability treatment weighting (IPTW) propensity score analysis. Journal of Biopharmaceutical Statistics.

• Zheng S, Wang D, Qiu J, Chen T, Gamalo M., 2023. A win ratio approach for

Authors

Ying Cui <cuiyingbeicheng@gmail.com>

Bo Huang
